# Why LGBT Teachers May Make Exceptional School Leaders

**DOI:** 10.3389/fsoc.2020.00050

**Published:** 2020-07-14

**Authors:** Catherine Lee

**Affiliations:** Faculty of Health, Education, Medicine and Social Care, Anglia Ruskin University, Cambridge, United Kingdom

**Keywords:** cis-normativity, school workplace, parents, heteronormativity, leadership, teachers, LGBT

## Abstract

The recent school gate protests about the inclusion of LGBT identities in the curriculum suggest that sexual identity remains an issue of moral panic in UK schools. Given this current climate, and the legacy of Section 28, schools have rarely been easy workplaces for LGBT teachers. For LGBT teachers, significant energy and vigilance is required then to navigate the heteronormative and cis-normative staffroom and classroom. There is evidence that LGBT teachers try to remain as invisible as possible in their schools so as to not draw attention to themselves (Lee, 2019a). Some avoid promotion to school leadership roles fearing that the status will necessitate greater personal scrutiny by school stakeholders. Based on key attributes including, reading people, compassion, and commitment to the inclusion of others, making connections managing uncertainty, courage, and risk-taking, this perspective piece argues that some of the strategies LGBT teachers deploy to manage the intersection of personal and professional identities in school equip them with an array of particular skills that are conducive to excellent school leadership.

## Introduction

This article argues that the strategies deployed by LGBT teachers to manage the intersection of their personal and professional identities equips them with a distinct set of skills that are valuable to effective school leadership. It begins by reflecting on the sociological and political landscape for LGBT teachers before considering five key attributes that LGBT teachers may acquire through their lived experience as an LGBT teacher. The article concludes by recognizing the value of specific leadership programmes that celebrate protected characteristics and stresses the importance for young people of diverse role models, committed teachers, and authentic school leaders.

## The Current Landscape

There are as many as 50,000 LGBT teachers in British schools, yet there are very few openly LGBT Headteachers (Lee, 2019a). The Equality Act of 2010 has done much to safeguard LGBT teachers from workplace discrimination but it is well-documented that despite advances in equalities legislation at the macro level, many LGBT teachers do not yet feel adequately protected or safe enough to be out to all stakeholders in their school workplaces (Gray, 2010; Lee, 2019a).

Schools remain woefully behind the majority of other workplaces when it comes to LGBT inclusion. This is because, since the advent of Ofsted and school league-tables, conservatism, and the approval of heterosexual, and conservative parents is at the heart of what schools do. Teachers are compelled to reflect the communities their schools serve through the Teachers Standards (Department for Education, 2013) which require that “personal beliefs are not expressed in ways which exploit pupils' vulnerability” and that teachers “must have proper and professional regard for the ethos, policies, and practices of the school in which they teach (p. 11).”

Schools remain entrenched in the biologically predetermined power-ridden categorisations of male and female (Gray, 2010). This is evident in all phases of compulsory education, from the toys available in the reception class home corner through to highly gendered expectations of school leavers at their prom (Robinson, 2002). Pupils are grouped or split for activities according to gender, and even amongst the staff, rigid binaries of male or female are evident from the way in which pupils are expected to address them as Mr, Mrs, or Miss.

Homophobia in schools is well-documented in UK Schools. Cocker et al. (2019) report that many LGBT families are compelled to adopt to quite elaborate strategies to navigate homophobic discourses in schools, and Carlile (2019) too posits that work must be done within primary schools to acknowledge and celebrate LGBT relationships.

According to Piper and Sikes (2010), when teachers stray into territories in which sexual and gender norms are explored or questioned, this has the potential to create moral panic. When heteronormativity is threatened then so too are the discourses of power in the school (Gray, 2010; Rudoe, 2010) and interventions usually follow. Section 28 of the Local Government Act (1988–2003) was one such state sanctioned intervention when local authorities (to which state schools at that time belonged) were forbidden from “the teaching in any maintained school of the acceptability of homosexuality as a pretended family relationship.” For 15 years, Section 28 created a climate in schools in which LGBT teachers feared they would lose their jobs should their LGBT identity be revealed in the school workplace. Worse still, for 15 years, young people were denied support for issues related to their sexual or gender identity, and children with same-sex parents were denied access to resources which featured families like theirs.

Despite the repeal of Section 28, the introduction of the Equality Act (2010) and the Equal Marriage Act (2015) there have in 2019, been school gate parental and faith group protests in Birmingham about the introduction of a new programme of Relationships, Sex, and Health Education that is inclusive of LGBT relationships. Anderton Park School was forced to go to the High Court seeking an injunction creating an exclusion zone around the school to prevent further protests, such was the devastating effect on pupils and staff. At nearby Parkfield School, the Assistant Headteacher, Andrew Moffatt received death threats for implementing resources that depicted LGBT characters. This raised anxiety for LGBT teachers with many equating the school gate protests with the hostilities of the Section 28 era.

Though rarely explicitly articulated, there is evidence that the principal fear of LGBT teachers is that the heteronormative school community will align their identity with discourses of hypersexuality and pedophilia [see Cavanagh (2008), Borg (2015), Thompson-Lee (2017)]. Piper and Sikes (2010) too observe that “fear of the pedophile taints adult–child relationships in general” (p. 567). Although Lee (2019b) suggests all teachers are potentially under suspicion, Piper and Sikes argue that “When the focus is on sex that is regarded as being outside of the norm the difficulties are magnified” (p. 567). As the title of the 2010 article by Piper and Sikes declares, “All Teachers are Vulnerable but Especially Gay Teachers” (p. 566).

It is not surprising then that LGBT teachers frequently report that significant energy, on top of an already demanding role, is needed to compartmentalize their personal and professional selves, vigilantly, and tentatively navigating the complexities of their heteronormative school communities and trying to remain as invisible as possible (Ferfolja, 2007). Invisibility in the school workplace is of course not conducive to job promotion (Rudoe, 2010) and many LGBT teachers avoid school leadership roles altogether. Leadership is inevitably accompanied by greater visibility in the school community and greater scrutiny and interest from school stakeholders, and for some LGBT teachers such intrusion is not worth the reward of a leadership role.

It can be argued however that the strategies LGBT teachers learn to deploy to navigate the complexities of the heteronormative and cisnormative school workplace equips them with a set of skills that are conducive to an exceptional and distinctly effective style of school leadership.

## Theoretical Framework

It is important to stress that this article rejects essentialist delineations of gender and sexuality recognizing that they perpetuate heteronormativity. In common with Butler (1990), this article recognizes that behaviors associated with gender and sexuality are “instruments of regulatory regimes” and “the normalizing categories of oppressive structures” (p. 13–14). When applied here, this article assumes that the oppressive structures of heteronormativity mean that some LGBT teachers experience the school workplace differently to their heterosexual and cis gendered colleagues. The behaviors needed to navigate the heteronormative school environment, when practiced over time equip LGBT teachers with particular abilities which give them a distinct set of skills and attributes.

## Leadership Attributes

There are five key leadership attributes that LGBT teachers may have in abundance. They are:

- Reading people
- Compassion and commitment to the inclusion of others
- Making connections
- Managing uncertainty
- Courage and risk-taking.

## Reading People

It is widely recognized that reading people is necessary for LGBT people to successfully negotiate heteronormative environments (Mîndru and Nǎstasǎ, 2017). Reading people is defined by De Melo et al. (2014) as “the ability to infer others” beliefs, desires, and intentions from their facial expressions (p. 1). They add that reading people is “important in interdependent decision making… about the others' intention to cooperate.”(p. 1).

LGBT people often develop highly developed instincts about the intentions of other people. Knight et al. (2014) show that gay men and lesbians tend to be disproportionately represented in occupations that require high levels social perceptiveness. This perception is practiced over time and often deployed at great speed to protect LGBT people from exposure to prejudice. Through extensive practice, LGBT teachers often need to become adept at reading people and situations, and horizon scanning to determine whether or not it is safe to be out. Every time an LGBT teacher enters a new environment, meets a new colleague or parent, they must be able to recognize the subtlest of dispositions and behaviors in others before judging whether or not where relevant, it is safe to acknowledge their sexual or gender identity, or whether it would be safer instead to espouse a neutral position or even to adopt a position of pseudo-cis gendered heterosexuality. When applied to leadership, being adept at reading people is a highly effective attribute. Snyder (2006) identified that LGBT leaders often develop excellent emotional intelligence becoming adept at reading people and situations. LGBT leaders who hone the skill of reading people are ideally placed to make good decisions when recruiting new employees (Snyder, 2006). They may also become astute and discerning when interacting with a wealth of different school stakeholders, especially heterosexual parents with traditional views on sexual and gender identity. This may include knowing how to engage difficult parents to diffuse an antagonistic situation and instinctively making good decisions on what information to share and what to withhold in the best interests of the school community.

## Compassion and Commitment to the Inclusion of Others

Although the Equality Act (2010) protects UK LGBT teachers from overt discrimination, equality does not necessarily ensure inclusion. Exclusion can be subtle, divisive, and oft times unintentional. LGBT teachers report extensive experience of feeling marginalized (Lee, 2019b). This may be in school staffrooms, within their wider community, through their families of origin and often within their own experiences as a pupil at school (Ryan et al., 2009). Having experienced exclusion and marginalization, LGBT teachers often have empathy in abundance and are more likely to be highly sensitized to inclusive best practice in their classrooms and amid teacher colleagues. Shallenberger (1994) asserted that the adversity endured through being othered by society, enabled gay men to develop an array of particular skills, valuable to leadership including sensitivity to diverse employees and an understanding of oppression. Brooks and Edwards (2009) observe that LGBT workers have three primary needs which are inclusion, safety, and equity. LGBT teachers are likely to have a heightened awareness of those on the margins of their school community and seek ways to ensure they feel included. When applied to leadership, LGBT teachers with personal experience of exclusion are likely to have developed a strong sense of social justice and an abundance of empathy with pupils, parents, and colleagues who may be marginalized on the basis of race, faith or social class, and other protected characteristics.

## Making Connections

This article has described the interminable heteronormativity and cis-normativity that stubbornly prevails in UK school communities. Within these conservative school workplaces LGBT teachers become skilful in identifying ways in which they can connect with others with whom they may not naturally have much in common. Alternative genders and sexualities are silenced in school communities to such an extent that the revelation of a same-sex partner is seen as belonging in the realm of the private and intimate, in the way that an opposite sex partner is not (Lee, 2019b). Fingerhut (2011) found that developing kinship and building alliances between LGBT and heterosexual allies is key to disrupting heteronormative spaces. LGBT teachers may then use the sharing of information deemed intimate to their advantage. By revealing their gender or sexual identity, they enter into subtle transactional discourses with cis and heterosexual colleagues, who often share information that belongs in the private realm in return (Hunter, 2007). The intimate sharing of personal information is invaluable for school leaders when building trust with different stakeholders across the school community. LGBT teachers are practiced at finding common ground with a diverse range of colleagues and stakeholders, and where LGBT school leaders are able to come out to colleagues, they report closer working relationships and greater levels of trust from their colleagues Studies by Bowring (2017), Jennings (2005), Leithwood and McAdie (2007), all concluded that LGBT educators being out contributed to a better environment for themselves, their colleagues, and their students. Being open about sexual identity fulfills a basic need to confirm and affirm one's identity. Disclosure allows individuals to form an authentic and stable sense of self (Rose Ragins, 2004), and reduces the cognitive dissonance and burden of identity management within the school workplace (King et al., 2008). LGBT teachers who enter into a “Don't ask, Don't tell” relationship with their school communities often feel that their personal identities are being silenced (Thompson-Lee, 2017).

## Managing Uncertainty

As this article has already posited, LGBT teachers are adept at tolerating a good deal of ambiguity and learn to function effectively when a great deal is uncertain. LGBT teachers often do not know for sure who knows about their gender or sexual identity. Space for declarative statements is often hard to find (Rasmussen, 2004) and rumor is usually commonplace in school communities. LGBT teachers present themselves to a host of different stakeholders in a variety of different contexts. This can be especially acute in rural school communities where there can be a blurring of the personal and professional (Lee, 2019a). Teacher colleagues are likely to be parents of children at the school and parents may be predominant members of the rural community known to the LGBT teacher in a different context (Thompson-Lee, 2017). Even when LGBT teachers are out to the entire school community they cannot be sure whether school stakeholders approve or privately hold homophobic beliefs and values (Khayatt, 1999). Amongst all this uncertainty, LGBT teachers learn to adopt a business as usual attitude, performing effectively and without distraction whilst inwardly often managing a considerable degree of personal turmoil, something Meyer (2003) identifies as minority stress. Most recently however, Meyer (2015) has observed that LGBT people can mount effective coping responses and most survive and even thrive despite minority stress. This is an exceptional skill for school leadership. School leaders often must protect their school communities from uncertainty, adversity, or bad news whilst exuding confidence, calm, and a sense of being in control. According to Hansen (2011) a crisis is always the true test of leadership. He states that whilst it is easy to lead well when things are going well, it is far more difficult when things are going poorly. LGBT teachers have extensive experience of operating under great personal stress whilst betraying nothing in their professional demeanor. This makes them ideally placed to provide composed leadership that reassures school stakeholders and builds trust in their leadership.

## Courage and Risk-Taking

Finally, courage and risk-taking are vital facets of school leadership and LGBT teachers often develop these in abundance. According to Snyder (2006), the gay leaders in his study were comfortable in risk taking, and using their non-conformity, became creative problem solvers because of their experience of having to create their own life paths in a heterosexist society. Each time LGBT teachers apply for a new position they must calculate whether or not their new school workplace will afford them the space to speak their authentic selves into existence, or whether instead they will be corralled into an all too common “Don't Ask, Don't Tell” (DePalma and Atkinson, 2009) arrangement with colleagues and line-managers. It is an act of considerable courage for an LGBT teacher to present themselves authentically within a new school workplace. Those commencing their careers during the Section 28 era know all too well that schools often provide no space for LGBT teachers to speak their identity into existence (Nixon and Givens, 2007). Creating such a space is an act of considerable courage and involves great personal risk to and may jeopardize future career prosperity (Rasmussen, 2004). The LGBT leaders in Shallenberger (1994) study, perceived themselves as more valuable to their employer because of their courage and willingness to take risks. Courage and calculated risk-taking is important in school leadership and LGBT teachers through years of risk-taking and acts of courage often build a heightened intuition that guides them in taking appropriate risks and demonstrating courage in the best interests of their school communities.

Few would disagree that in order to flourish educationally, young people need access to diverse role models, committed teachers, and authentic school leaders (Lee, 2019a). When LGBT leaders become visible within their school communities, they embody a distinct and exceptional type of leadership (Fassinger et al., 2010) through the acquisition and application of the five attributes identified in this article. When LGBT teachers become school leaders, they trouble institutional heteronormative and heterosexist practices (Gray, 2013) and via their own visibility, give other school stakeholders such as children and young people, parents, and colleagues, permission to also participate authentically and without fear. At a time when the average length of service of a Headteacher is just 3 years, it is crucial that investment into effective and distinct school leadership programmes (e.g., Courageous Leaders, LGBTEd, as well as Women Ed and BAME Ed) continues, so that we attract, recruit, and keep talented school leaders who reflect the full diversity of British society.

## Author Contributions

The author confirms being the sole contributor of this work and has approved it for publication.

## Conflict of Interest

The author declares that the research was conducted in the absence of any commercial or financial relationships that could be construed as a potential conflict of interest.
